# Metagenomic Exploration of Koumiss from Kazakhstan

**DOI:** 10.1128/mra.01082-21

**Published:** 2022-01-06

**Authors:** Andrey Bogoyavlenskiy, Madina Alexyuk, Pavel Alexyuk, Makhabbat Amanbayeva, Elmira Anarkulova, Anar Imangazy, Aliya Bektuganova, Vladimir Berezin

**Affiliations:** a Abai Kazakh National Pedagogical University, Almaty, Kazakhstan; b Research and Production Center for Microbiology and Virology, Almaty, Kazakhstan; Loyola University Chicago

## Abstract

Here, we report a metagenomic analysis of koumiss from Kazakhstan. In this study, shotgun metagenomic sequencing of the RNA and DNA viral community was performed.

## ANNOUNCEMENT

Bacterial infections of the lung and gastrointestinal tract remain one of the main problems of medicine in a number of countries (1–3). The emergence of antibiotic-resistant variants of microorganisms has led to the need to search for new nonstandard methods of treating bacterial infections. One of these methods is koumiss treatment of pulmonary and gastrointestinal infections in regions with developed horse breeding. It was shown that the composition of koumiss is of great importance for the additional treatment of microbial diseases (4, 5). In our research, the virome of koumiss (fermented dairy product made from mare’s [Equus caballus] milk) was studied to diagnose the presence of phages in this dairy product that can lyse pathogenic microorganisms, creating a therapeutic effect. Koumiss prepared on meat and dairy cattle farms in Talgar near Almaty (43°18′N, 77°14′E) was used for the study.

A shotgun metagenomic study of the DNA and RNA viral community, including sample preparation, sequencing, and bioinformatics analysis, was mainly performed as described in our previous studies (6–8). Briefly, 2 L koumiss was centrifuged at 15,000 rpm for 15 min at 4°C, filtered using a 0.45-mm membrane to remove most of the bacteria, and concentrated by ultracentrifugation using a Beckman Coulter Avanti J30I ultracentrifuge at 29,000 rpm for 2 h at 4°C. Total DNA was isolated using a PureLink genomic DNA extraction kit (Thermo Fisher Scientific, USA) and stored at 2 to 8°C. Viral RNA was extracted using a QIAamp viral RNA minikit (Qiagen). The sample extracts were pretreated with RNase-free DNase (Promega). Double-stranded cDNA was obtained using a SuperScript double-stranded cDNA synthesis kit (Invitrogen) according to the manufacturer’s instructions. Genomic DNA and synthesized double-stranded cDNA were pooled. The paired-end library was prepared using a Nextera XT DNA sample preparation kit (Illumina, USA) according to the manufacturer’s protocol. Sequencing of the library was conducted on the Illumina MiSeq platform using the MiSeq reagent kit v3 (2 × 300-bp cycles; Illumina). The obtained sequences (2,719,939 reads) were tested for quality using FastQC v0.11.9 (https://www.bioinformatics.babraham.ac.uk/projects/fastqc) and Trimmomatic v0.36 (9) within the Genome Detective tool (10). Reads with average quality of less than 20 and length less than 200 bp were removed. Taxonomic identification of viral sequences was performed by Kaiju using the BLASTn v2.5.0 algorithm (10) against the NCBI RefSeq viral complete genome database (26 February 2021; 20 GB) (11). Default parameters were used for all software. The sequence reads were considered “identified” if they had a relative in the reference database with an E value of <10^−5^ and a bit score of >50.

**FIG 1:**
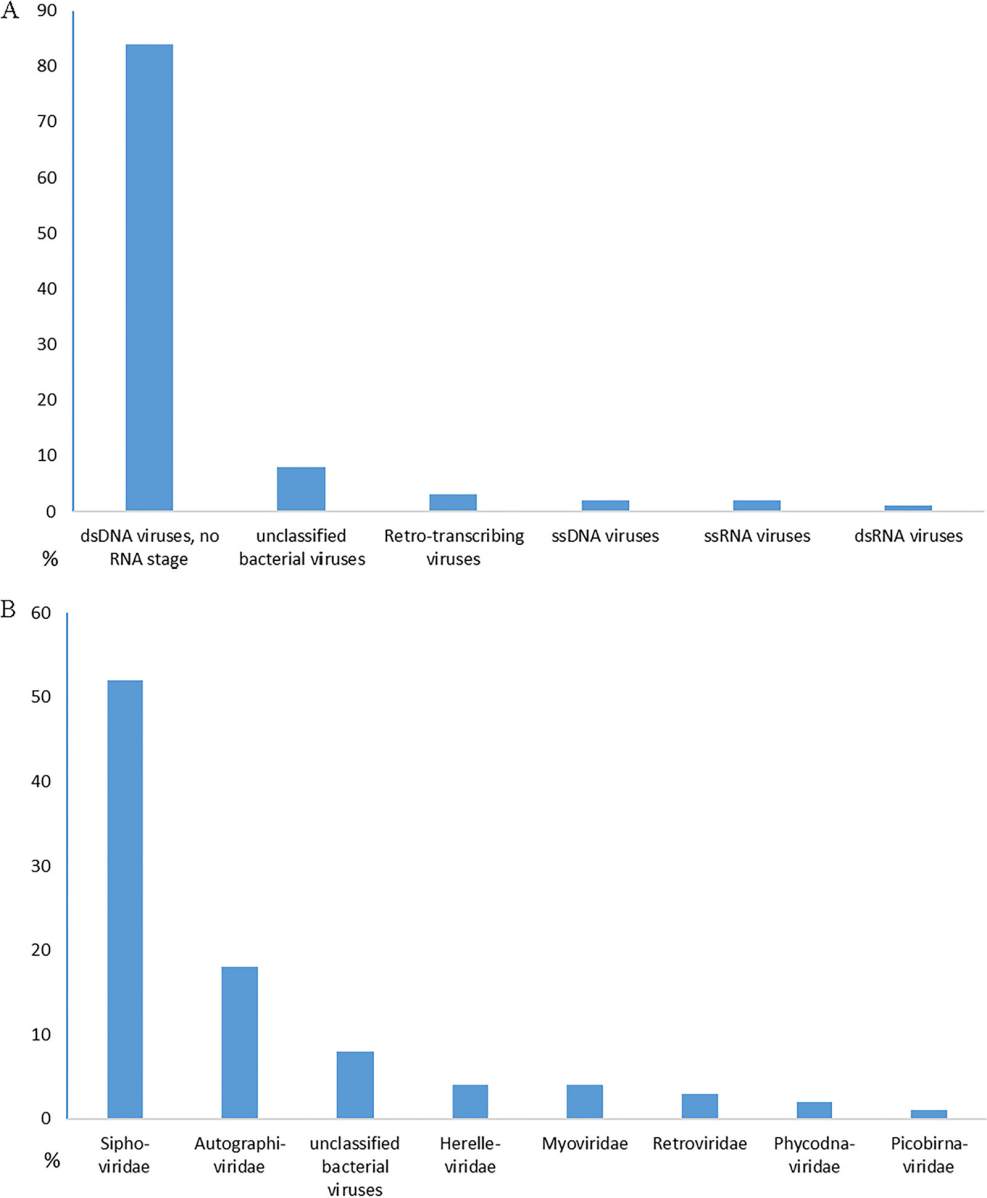
(A) Viral groups of the koumiss virome. (B) The main viral families of the koumiss virome. Microsoft Excel 2016 was used to generate the plots.

The raw data contained 2,719,939 paired sequence reads. After quality processing, we obtained 2,033,388 single reads; of these, 573,066 sequences (28.2% of the data set) were identified as viral, belonging to double-stranded DNA (dsDNA) viruses with no RNA stage (84%), retro-transcribing viruses (2%), single-stranded DNA (ssDNA) viruses (2%), ssRNA viruses (2%), dsRNA viruses (1%), and unclassified bacterial viruses (8%) (Fig. 1A). Despite the fact that representatives of 70 families of viruses were detected, 7 of them comprised 92% of all identified viruses (Fig. 1B). In total, we revealed a high viral diversity in the samples of koumiss (721 virotypes from 70 families). Among these viral groups, viruses were found that are able to lyse the main groups of microorganisms that cause diseases of the lungs and gastrointestinal tract. Among these microorganisms are those belonging to the genera Streptococcus, Mycobacterium, Campylobacter, Pseudomonas, Acinetobacter, etc.

Our metagenomic research allowed us to start exploring the diversity of viral communities in koumiss, which will make it possible to assess the role of koumiss in traditional medicine.

### Data availability.

The raw sequence reads are available under BioProject accession number PRJNA775816.
